# Case Report: Clinical application of continuous arterial infusion chemotherapy in neoadjuvant therapy for locally advanced gastric cancer

**DOI:** 10.3389/fonc.2023.1214599

**Published:** 2023-06-23

**Authors:** Wenli Lin, Zhongxian Huang, Zhenhua Du, Yunshan Wang, Taiyang Zuo

**Affiliations:** ^1^ Department of Interventional Oncology, Central Hospital Affiliated Shandong First Medical University, Jinan, Shandong, China; ^2^ Department of Urology, Central Hospital Affiliated Shandong First Medical University, Jinan, Shandong, China; ^3^ Basic Medical Research Center, Central Hospital Affiliated Shandong First Medical University, Jinan, Shandong, China

**Keywords:** continuous arterial infusion, intra-arterial infusion, locally advanced gastric cancer, neoadjuvant therapy, chemotherapy

## Abstract

Platinum-fluorouracil combination chemotherapy is the standard neoadjuvant treatment for locally advanced gastric cancer in China, but it does not improve the survival benefit of patients. In recent years, the application of immune checkpoint inhibitors and/or targeted drugs in neoadjuvant therapy for gastric cancer has achieved certain efficacy, but the survival benefit of patients is still not obvious. Intra-arterial infusion chemotherapy, as a method of regional therapy, has been widely used in the treatment of many advanced tumors and achieved remarkable curative effect. The role of arterial infusion chemotherapy in neoadjuvant therapy for gastric cancer is not clear. We describe two patients with locally advanced gastric cancer treated with continuous arterial infusion neoadjuvant chemotherapy. Two patients received continuous arterial infusion of chemotherapy drugs for 50 hours, the drugs were pumped into the main feeding artery of the tumor through the arterial catheter. A total of 4 cycles were followed, then undergone surgical resection. The postoperative pathological pCR of two patients was 100%, TRG was 0 grade, and no further anti-tumor therapy was required after operation, achieving clinical cure. During the treatment period, no serious adverse events occurred in either patient. These results suggest that continuous arterial infusion chemotherapy may be a new adjuvant therapy for locally advanced gastric cancer.

## 1 Introduction

The incidence and mortality of gastric cancer account for the third place among all kinds of malignant tumors in China in 2020. Only low percentage (about 20%) of gastric cancers are diagnosed in its early stage. Most of patients were in advanced stage, among which the proportion of locally advanced gastric cancer (LAGC)T3/4,N+, M0) reached a new high of 70.8% (1). The standard treatment regimen of for LAGC is radical resection or neoadjuvant chemotherapy (NAC) followed by radical resection (2). In China, 40% - 50% of patients with LAGC could receive radical resection, but the recurrence rate after radical resection alone was as high as 80%, the overall survival (OS) was about 12 months, and the 5-year survival rate was only about 35.9% (3). In order to achieve better therapeutic efficacy, more attention should be paid to the breakthrough progress in neoadjuvant therapy. Neoadjuvant therapy can not only reduce the tumor stage, improve the surgical resection rate and radical resection rate, but also reduce the risk of tumor recurrence and metastasis (4, 5), so it is recommended as one of the standard treatment modalities for LAGC (2). However, the indications, regimens, and cycles of neoadjuvant therapy are not strictly defined, and the impact of neoadjuvant chemotherapy on the survival of patients with gastric cancer *in situ* is still controversial (6). So, the clinical application of neoadjuvant chemotherapy is limited. The proportion of LAGC patients receiving neoadjuvant therapy in China is only 13.8%, and the pathologic complete remission (pCR) rate after neoadjuvant therapy was only 11.4% (7). In order to break the current predicament of LAGC treatment, scholars have made many attempts in recent years, especially in neoadjuvant therapy. Some scholars have added immune checkpoint inhibitors to the neoadjuvant therapy of gastric cancer (8), hoping to improve the therapeutic efficacy for gastric cancer by changing the types of drugs used in neoadjuvant therapy. Some scholars have also explored the therapeutic effect of immune checkpoint inhibitors combined with chemotherapy-targeted drugs on special types of gastric cancer (such as mismatch repair deficiency, dMMR/high microsatellite instability,MSI-H) (9), trying to further optimize the treatment regimen from different subtypes of gastric cancers. Sintilimab combination with neoadjuvant therapy for LAGC resulted in 19.4% of pCR, and 94.1% of one-year OS (10). Neoadjuvant therapy of Apatinib plus chemotherapy for gastric cancer (11) showed median event-free survival was 42 months. Unfortunately, most of the recent studies were phase II clinical studies, which need validation from phase III clinical trials. In addition, neoadjuvant chemotherapy of multi-agent combination did not significantly improve the overall survival of patients. Therefore, the treatment of LAGC still faces great challenges, and new treatment modes or strategies are urgently needed.

The key of LAGC treatment is to improve the local tumor control rate. Which can be improved by increasing the local drug concentration. Intra-arterial infusion chemotherapy is one way to increase regional drug concentration. This way is to infuse chemotherapy drugs directly into the tumor feeding artery through catheter. Compared with intravenous chemotherapy, intra-arterial chemotherapy can increase the drug concentration in tumor tissue by 2 - 4 times (12). The regional high concentration of the drug can ensure that the chemotherapeutic drug exert the maximum anticancer effect and improve the curative effect of tumor treatment. Arterial infusion chemotherapy is currently widely used in advanced liver cancer (13–15), advanced pancreatic cancer (16), etc. Some scholars have preliminarily explored the efficacy and safety of intra-arterial infusion of oxaliplatin combined with oral S-1 as neoadjuvant therapy for LAGC. The results showed that 4 patients had no obviously adverse events and the tumor regression rate reached to 100% (17). It is suggested that intra-arterial infusion chemotherapy can be used as a new adjuvant therapy for LAGC, which is worth further study. Here, we reported two patients with LAGC who received neoadjuvant therapy via continuous arterial infusion (50 hours). Postoperative pathology showed no tumor cells and tumor regression grading (TRG) (18) was 0. In addition, both patients did not receive any anti-tumor therapy after operation.

## 2 Case presentation

### 2.1 Case 1

**Figure 1:**
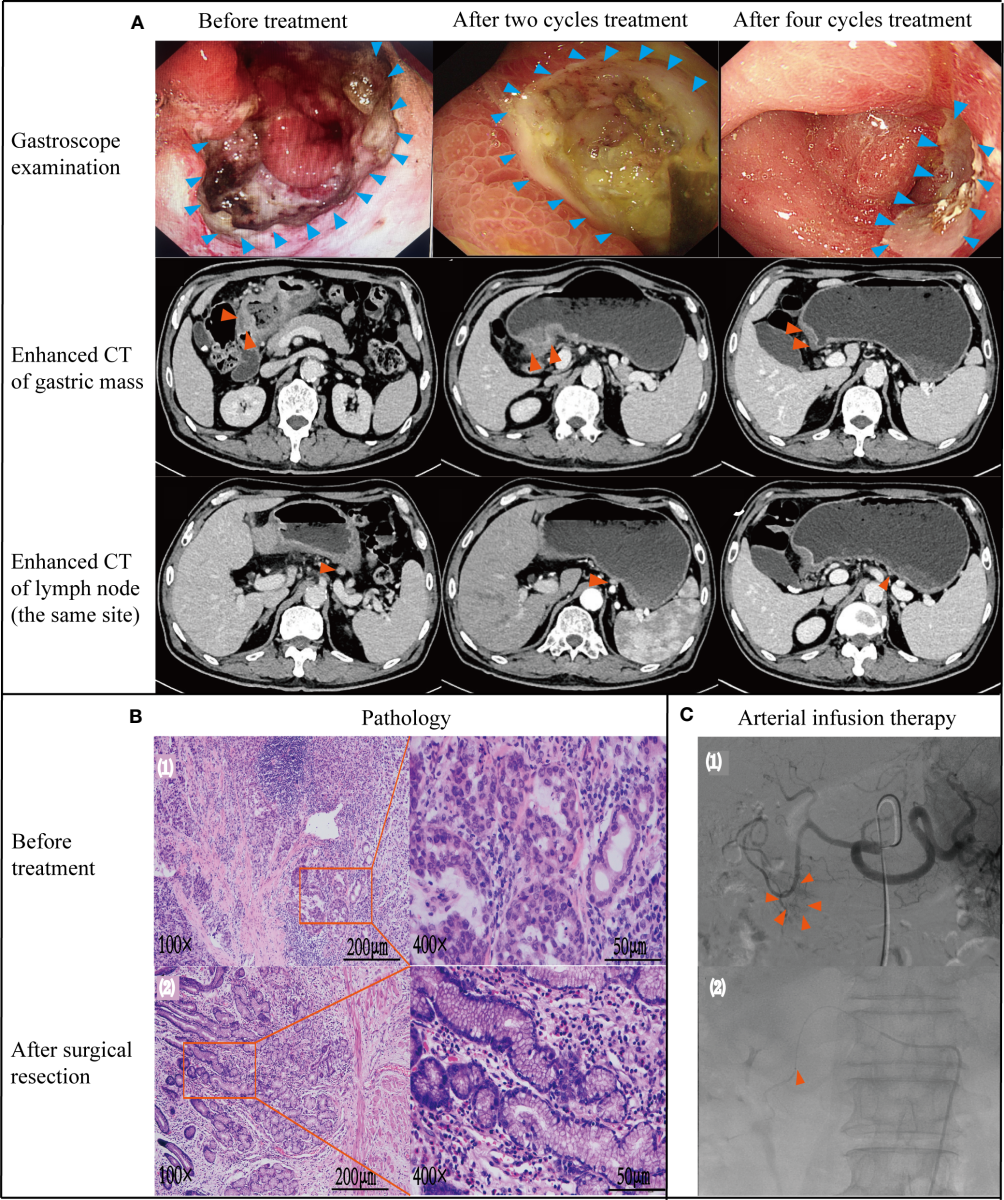
Case data of patient 1. **(A)** Gastroscope and imaging examination.Changes of tumor or lymph node in gastroscopy and enhanced CT before treatment, after 2 and 4 cycles treatment. **(B)** Pathology examination (1): biopsy pathology before treatment (adenocarcinoma);(2): pathology after surgical resection (no tumor cells found) **(C)** Treatment process under DSA (1): angiography showed that the feeding artery of the tumor was gastroduodenal artery; (2): microcatheter tip placement location (in gastroduodenal artery).

A 74-year-old male was admitted to the local hospital due to right upper abdominal pain and discomfort for half a year, accompanied by weight loss of 6kg. Gastroscopy revealed a large ulcerated lesion of the antral mucosa, with annular growth, fragile texture and bleeding (**Figure 1A**). Biopsy pathology diagnosis showed poorly differentiated adenocarcinoma [**Figure 1B** (1)]. The patient visited our hospital for further treatment on April 23th,2021. Body weight at admission was 68kg, hemoglobin (Hb) level was 92g/L (normal reference value:130~175g/L), stool occult blood was negative. The levels of CEA\CA199\AFP\CA724 were within normal limits. Enhanced CT of the abdomen showed the presence of metastases in regional lymph node (**Figure 1A**) but no distant metastases. Clinical staging was performed using the AJCC/UICC TNM staging system for gastric cancer, eighth edition (19), and the stage was cT3N3M0, stage III. After discussion by the multidisciplinary team, the radical resection was feasible for this patient, but the patient and his family refused the treatment suggestion. With the consent of patient and his family, arterial infusion neoadjuvant therapy was started on 30-April-2021.

DSA showed that the gastroduodenal artery was the main feeding artery of the tumor [(**Figure 1C** (1)]. The microcatheter was placed in the gastroduodenal artery under DSA guidance [(**Figure 1C** (2)]. Specific dosage and usage of drug pumped through indwelling arterial catheter: Oxaliplatin (70mg/m2) pumped for 2h; Calcium folinate (200mg/m2) pumped for 2h; Fluorouracil (1600mg/m2) pumped for 46h. 28 days was a course of treatment. Four cycles were performed.

**Figure 2:**
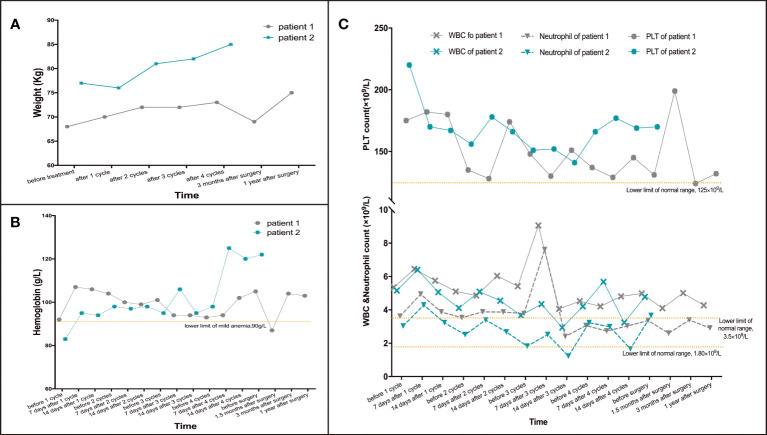
Changes of weight and blood cells in patient 1 and 2 during treatment. **(A)** Changes of weight during treatment. **(B)** Changes of hemoglobin during treatment. **(C)** Changes of PLT, WBC& neutrophils during treatment.

After 2 cycles of treatment, the patient’s upper abdominal pain disappeared, with weight of 72kg and Hb of 101g/L. Enhanced abdominal CT and gastroscopy revealed a significant reduction in tumor volume (**Figure 1A**). According to the evaluation criteria for response in solid tumors, RECIST 1.1 criteria (20), efficacy was evaluated as partial response (PR). Reexamination after 4 cycles of arterial infusion treatment showed that the body weight was 73kg and Hb was 105g/L. Enhanced CT and gastroscopy of abdomen showed further reduction of tumor volume (**Figure 1A**), and the efficacy evaluation was PR again. 6 weeks after arterial infusion therapy. On September 18^th^,2021, laparoscopic distal subtotal gastrectomy & D2 lymphadenectomy was performed. Postoperative pathology showed no tumor cells in gastric ulcer and lymph nodes [**Figure 1B** (2)], TRG grade was 0. No anti-tumor therapy was given after operation, and the patient has regular followed-up with doctors till now. The patient experienced no significant adverse reactions throughout the treatment (**Figure 2**). The Hb increased to 105 g/L one year postoperatively and the weight was 78 kg. Changes in weight and blood cells during treatment were shown in (**Figure 2**.

### 2.2 Case 2

**Figure 3:**
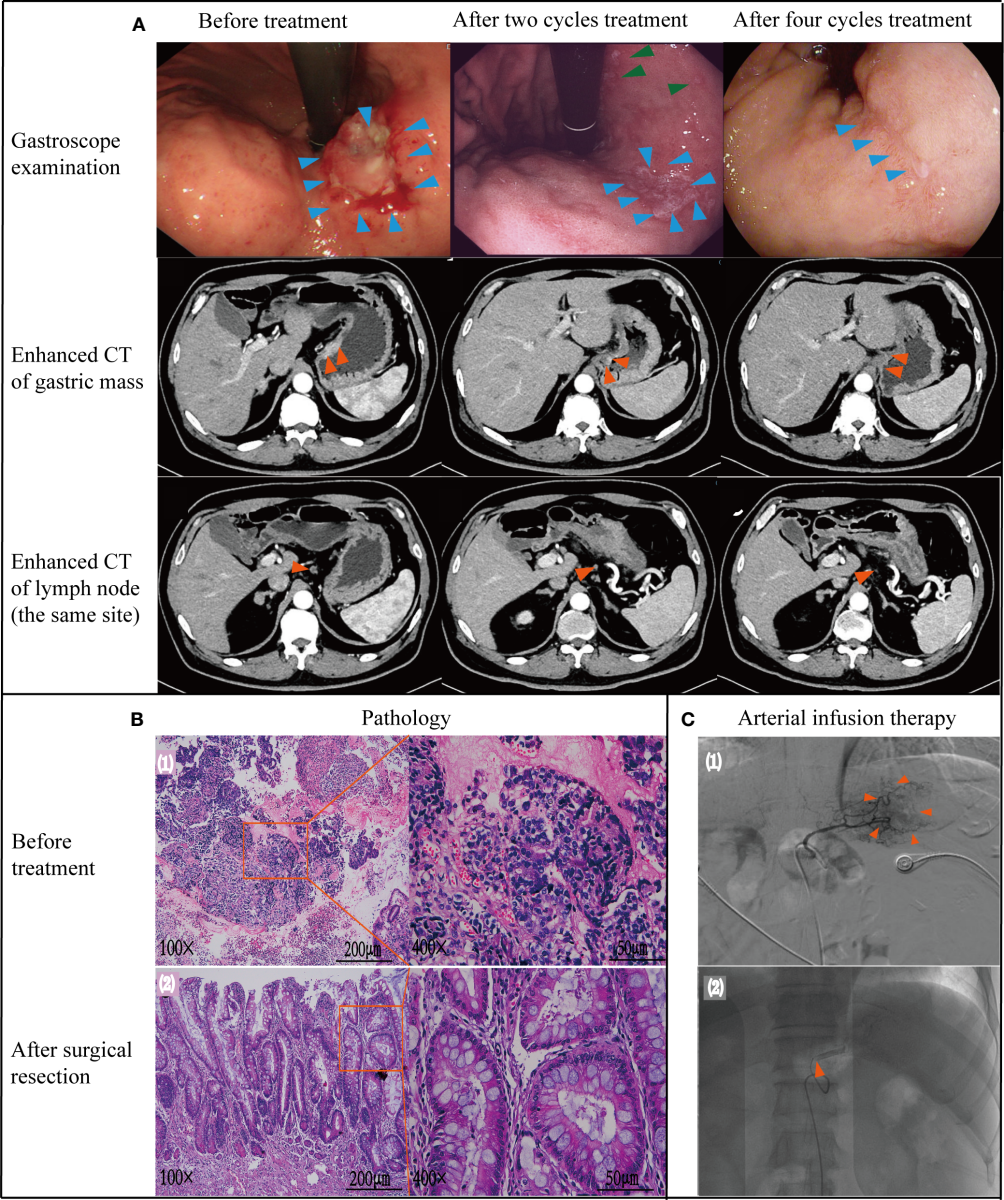
Case data of patient 2. **(A)** Gastroscope and imaging examination. Changes of tumor or lymph node in gastroscopy and enhanced CT before treatment, after 2 and 4 cycles treatment. **(B)** Pathology examination (1): biopsy pathology before treatment (adenocarcinoma); (2): pathology after surgical resection (no tumor cells found). **(C)** Treatment process under DSA (1): angiography showed that the feeding artery of the tumor was left gastric artery; (2): microcatheter tip placement location (in left gastric artery) Note: Figure 3A - green arrows. After 2 cycles of treatment, gastroscope also showed multiple small ulcers in the gastric body of patient 2, which was considered to be gastric mucosal reaction caused by high-concentration chemotherapy drugs in arterial infusion chemotherapy. After treatment with proton pump inhibitors, the patient's multiple ulcers improved and healed.

A 66-year-old man with a history of hypertension and diabetes mellitus, visited our hospital due to anemia for 2 months with weight loss of 4kg on Dec 24^th^, 2021. Weight on admission was 77kg. Gastroscopy showed ulcerative neoplasia about 3cm×4cm in size on the posterior wall of lesser curvature of stomach, hyperemia and erosion, brittle and easy to bleed (**Figure 3A**). Biopsy pathology diagnosis was showed adenocarcinoma [**Figure 3B** (1)]. Hb level was 83 g/L, stool occult blood was positive. The levels of CEA\CA199\AFP\CA724 were within normal limits. Abdominal enhanced CT showed the presence of metastasis in regional lymph node but no distant metastasis (**Figure 3A**), and the clinical stage was cT3N2M0, stage III. The patient and his family also refused the preferred surgical resection. Arterial infusion neoadjuvant therapy was started on Jan 10^th^, 2022 with the consent of patient and his family.

DSA showed that the left gastric artery was the main feeding artery for the tumor [**Figure 3C** (1)]. Under DSA guidance, a microcatheter was placed in the left gastric artery [**Figure 3C** (2)], and chemotherapy drugs were pumped through the indwelling arterial catheter. The specific drug dosage and usage are the same as those in Case 1. Reexamination after 2 cycles revealed Hb was 98 g/L and weight was 81 kg. Enhanced CT and gastroscopy of the abdomen showed a significant reduction in tumor volume and irregular superficial ulceration (**Figure 3A**). Efficacy was evaluated as PR. During gastroscopy after 2 cycles of treatment, the patient found multiple small ulcers in the gastric body, which was considered to be gastric mucosal reaction caused by high-concentration chemotherapy drugs in arterial infusion chemotherapy. After treatment with proton pump inhibitors, the patient’s multiple ulcers improved and healed (**Figure 3A**, green arrows).

Reexamination after 4 cycles of arterial perfusion showed that the weight was 85 kg and Hb was 99 g/L. The imaging findings showed further tumor shrinkage (**Figure 3A**), and the efficacy evaluation was PR. After 6 weeks of arterial infusion therapy, laparoscopic distal subtotal gastrectomy & D2 lymphadenectomy was performed on May 11^th^,2022. Postoperative pathology showed no tumor cells in gastric ulcer and lymph nodes [(**Figure 3B** (2)], TRG grade was 0. No anti-tumor treatment was given after the operation for this patient. However the patient died due to accident and was lost to follow-up. Throughout the treatment, the patient had grade I myelosuppression, and resolved spontaneously without special treatment (**Figure 2**). Hb level increased to 122 g/L postoperatively. Changes in weight and blood cells during treatment were shown in (**Figures 2**.

## 3 Discussion

The incidence of gastric cancer varies widely among regions of the world, with the highest incidence in East Asian populations and lower in North American populations (21, 22). China has the highest incidence of gastric cancer in East Asia. In 2019, the number of gastric cancer patients in China accounted for 44.21% of total GC cases in East Asia (23), and most patients were in advanced stage at diagnosis. It can be said that gastric cancer is a disease with Chinese characteristics, mainly characterized by late onset and late stage. In addition, the 5-year survival rate of gastric cancer in China was only 35%~40% (1). So, the treatment of gastric cancer in China is very challenging. Nearly 50% of patients still relapsed after surgery plus the standard regimen of perioperative platinum-based chemotherapy (24). Scholars attempted to get rid of the predicament of neoadjuvant chemotherapy for gastric cancer by adding new drugs. For example, Nivolumab plus Ipilimumab were applied to the combination regimen of neoadjuvant chemotherapy for the treatment of locally deficient mismatch repair/microsatellite instability high gastric or esophagogastric junction adenocarcinoma. The results showed that 60% of patients had pCR and 19% of patients had grade 3 - 4 adverse events (9). Sintilimab was added to platinum-based neoadjuvant chemotherapy, only 19.4% of patients achieved pCR (10). Therefore, the progress of neoadjuvant therapy for gastric cancer is still slow, and it is still inconclusive for treatment regimen and cycle.

Arterial infusion chemotherapy belongs to regional chemotherapy, which is essentially a pharmacokinetic treatment of drug delivery into solid malignancies (12). Arterial infusion chemotherapy can make more concentrated drug in the tumor target area. Arterial infusion chemotherapy has its unique theoretical advantages in the treatment of tumors: 1) Arterial infusion chemotherapy has strong pertinence (targeting). The drugs can be accurately located in the tumor feeding artery. High-concentration drug directly acts on the tumor part without metabolism, and the drug concentration in the local tumor tissue reaches tens to hundreds of times that of the normal tissue, so that the anti-tumor effect can be maximized. Previous studies of HAI showed that the exposure of FUDR, 5-fu, and DDP in tumor tissue increased by 100-to 400-fold, 5-to 10-fold, and 4-to 7-fold in the hepatic arterial route compared with the intravenous route (25). 2) High-concentration drugs mainly act on local tumor, and the drug concentration flowing through other tissues and organs of the whole body is low, which reduces the damage of chemotherapy drugs on other tissues, thus protecting other normal tissues, explaining the relatively low incidence of adverse reactions of arterial infusion chemotherapy (16, 26, 27). Clinical studies had demonstrated that hepatic arterial infusion chemotherapy can significantly increase the rate of resectable transformation in patients with colorectal liver metastases (28). The median OS of advanced unresectable intrahepatic cholangiocarcinoma was only 11 months, and the control rate of patients with advanced unresectable intrahepatic cholangiocarcinoma was 84% with a median OS of 25 months (29). However, there is no report about the continuous arterial infusion chemotherapy in neoadjuvant therapy of gastric cancer. The patients in this study were treated with neoadjuvant therapy by arterial infusion. Intra-arterial infusion chemotherapy is the same as systemic chemotherapy, but only changes the route of administration. In this study, the dose of continuous arterial infusion was slightly lower than that of intravenous infusion, but significant curative effect was still achieved. The TRG classification of 2 patients with LAGC was 0, and no other special treatment was required after operation. The survival and quality of life of patients were greatly improved. It is suggested that intra-arterial infusion chemotherapy was effective in the neoadjuvant therapy of LAGC. It is worth mentioned that the related adverse reactions caused by chemotherapy drugs are reduced, due to the low dose of drugs used during arterial infusion chemotherapy. Only one of the 2 patients presented in this paper had grade I bone marrow suppression, which recovered spontaneously without special treatment. It was suggested that the safety of arterial infusion chemotherapy was high.

In this study, patient 2 was found to have multiple new small ulcers in gastric body during gastroscopy after 2 cycles of treatment, which was considered to be gastric mucosal reaction caused by high-concentration chemotherapy drugs by arterial infusion. After treatment with proton pump inhibitor, the patient’s multiple ulcers improved and healed. It was suggested that the high concentration of chemotherapeutic drugs may lead to ulcer and other complications in normal gastric mucosa during arterial infusion therapy. Previous literature has reported that the incidence of arterial thrombosis associated with arterial cannulation may be high (28). There were no adverse reactions related to arterial cannulation in either patient in this study. This article reports a new neoadjuvant therapy for LAGC, continuous intra-arterial infusion chemotherapy, which shows good tolerability, safety and significant therapeutic efficacy.

## Data availability statement

The original contributions presented in the study are included in the article/supplementary material. Further inquiries can be directed to the corresponding author.

## Ethics statement

The studies involving human participants were reviewed and approved by Ethics Committee of Central Hospital affiliated Shandong First Medical University. The patients/participants provided their written informed consent to participate in this study. Written informed consent was obtained from the individual(s) for the publication of any potentially identifiable images or data included in this article.

## Author contributions

WL, YW and TZ contributed to conception and design of the study. ZD and ZH organized the database and performed the statistical analysis. WL wrote the first draft of the manuscript. YW and TZ wrote sections of the manuscript. All authors contributed to manuscript revision, read, and approved the submitted version. All authors contributed to the article and approved the submitted version.
